# Evaluation of 24-Hour Arterial Stiffness Indices and Central Hemodynamics in Healthy Normotensive Subjects versus Treated or Untreated Hypertensive Patients: A Feasibility Study

**DOI:** 10.1155/2015/601812

**Published:** 2015-01-26

**Authors:** Stefano Omboni, Igor N. Posokhov, Anatoly N. Rogoza

**Affiliations:** ^1^Clinical Research Unit, Italian Institute of Telemedicine, 21048 Solbiate Arno, Italy; ^2^Hemodynamic Laboratory, P.O. Box 69, Nizhny Novgorod 603009, Russia; ^3^Department of New Methods of Diagnostics, Russian Cardiology Research and Production Complex, Moscow 121552, Russia

## Abstract

*Objective*. Central blood pressure (BP) and vascular indices estimated noninvasively over the 24 hours were compared between normotensive volunteers and hypertensive patients by a pulse wave analysis of ambulatory blood pressure recordings. *Methods*. Digitalized waveforms obtained during each brachial oscillometric BP measurement were stored in the device memory and analyzed by the validated Vasotens technology. Averages for the 24 hours and for the awake and asleep subperiods were computed. *Results*. 142 normotensives and 661 hypertensives were evaluated. 24-hour central BP, pulse wave velocity (PWV), and augmentation index (AI) were significantly higher in the hypertensive group than in the normotensive group (119.3 versus 105.6 mmHg for systolic BP, 75.6 versus 72.3 mmHg for diastolic BP, 10.3 versus 10.0 m/sec for aortic PWV, −9.7 versus −40.7% for peripheral AI, and 24.7 versus 11.0% for aortic AI), whereas reflected wave transit time (RWTT) was significantly lower in hypertensive patients (126.6 versus 139.0 ms). After adjusting for confounding factors a statistically significant between-group difference was still observed for central BP, RWTT, and peripheral AI. All estimates displayed a typical circadian rhythm. *Conclusions*. Noninvasive assessment of 24-hour arterial stiffness and central hemodynamics in daily life dynamic conditions may help in assessing the arterial function impairment in hypertensive patients.

## 1. Introduction

In recent years, great emphasis has been placed on the role of arterial stiffness and central blood pressure (BP) as independent predictors of the development of cardiovascular (CV) diseases [1–3]. Consequently, the assessment of arterial stiffness and central hemodynamics is recommended as additional tests for the clinical evaluation of hypertensive patients (based on history, physical examination, and findings from routine laboratory tests), particularly for those at risk of CV complications [4].

Regional and local arterial stiffness may be measured directly and noninvasively, at various sites along the arterial tree, by assessing pulse wave velocity (PWV) and augmentation index (AI) [1]. Central BP is derived from noninvasive techniques of measurement of radial or carotid pulses [5].

The most widely employed methods for evaluating pulse waveforms are those based on applanation tonometry and transfer functions, although recently oscillometric ambulatory blood pressure monitoring (ABPM) devices using specific algorithms for pulse wave analyses have been proposed for assessing arterial stiffness [6–9]. At present, oscillometry is an affordable technique and may allow a comfortable, accurate, repeated, and prolonged estimation of arterial stiffness and central hemodynamics over the 24 hours in daily life conditions [9]. The most recent studies seem to indicate reliability and feasibility of ambulatory arterial stiffness evaluation based on analysis of brachial oscillograms [10, 11].

In the present study we aimed at assessing the feasibility of determining central BP and various indices of arterial stiffness over the 24 hours by a noninvasive, clinically validated technology of pulse wave analysis based on oscillometric BP measurements, integrated in an ambulatory BP (ABP) monitor [10, 12]. Potential differences in arterial hemodynamics and stiffness were sought between healthy normotensive volunteers and hypertensive patients evaluated in a real-life context.

## 2. Materials and Methods

### 2.1. Study Population and Design

Treated or untreated hypertensive outpatients and untreated healthy volunteers, aged 18 years or more, were included in the study. Healthy individuals were eligible for inclusion into the study in absence of arterial hypertension (office systolic, SBP < 140 mmHg, and office diastolic, DBP < 90 mmHg plus 24-hour average SBP < 130 mmHg and DBP < 80 mmHg), blood test abnormalities (including impaired fasting glucose, impaired glucose tolerance, or dyslipidemia), obesity, and other major cardiovascular risk factors. Both healthy subjects and hypertensive patients were excluded in case of previous or current cardiovascular disease or any other concomitant significant systemic condition. All individuals were submitted to an ABPM, preceded by an office automatic BP measurement with the same device used for ambulatory monitoring. Office BP was measured in the sitting position after 5-minute rest: three measurements were obtained at 2 min intervals and the average of the three measurements was taken as the reference for office BP.

Hypertensive patients were recruited among consecutive patients with a known history of high BP presenting at the outpatient clinic of the Cardiology Research Complex, Moscow. Healthy volunteers were recruited among the staff and personnel at the Russian Railroad and at the Russian Navy. The study was conducted according to Good Clinical Practice guidelines and the Declaration of Helsinki, and the protocol was approved by the ethics committees of the centers involved. Written informed consent was obtained from all patients and controls prior to their inclusion into the study. All individuals were recruited and studied between September 2008 and December 2012.

### 2.2. ABP Measurement

ABPM was performed noninvasively over the 24 hours by the BPLab electronic, oscillometric, automated BP monitor (BPLab Gmbh, Germany). The device accuracy in measuring BP has been previously successfully tested in a validation study [13]. Additionally, the device has passed validation also for estimation of vascular indices against the most commonly noninvasive device, recommended as reference standard, the SphygmoCor [12, 14], in accordance with the ARTERY guidelines [15].

Current international guidelines were followed for proper ambulatory recording performance [16]. The optimal adult cuff was wrapped around the nondominant arm and the patient was asked to keep her/his arm still during the automatic BP measurements. The device was programmed to measure BP every 15–30 min during daytime (from 06:00 to 22:00) and every 30–60 min during nighttime (from 22:00 to 06:00). Each recording started in the morning and was preceded by verification of the accuracy of oscillometric BP measurements against auscultatory technique in every subject. After fitting the device, patients were sent home and asked to resume normal life and to come back 24 hours later for removal of the instrumentation.

### 2.3. Measurement of Ambulatory Arterial Stiffness and Central Hemodynamics

The BPLab BP monitor makes use of brachial oscillometric BP waves for a noninvasive estimation of central BP and arterial stiffness [10, 12–14]. The Vasotens principle of oscillometric pulse wave analysis is based on plethysmography and on recording the pulsatile pressure in the brachial artery. During BP measurement, the pressure waveforms in the cuff are digitalized and stored in the device memory while performing step-by-step deflation. Thereafter, signal processing is performed using a special mathematical algorithm, which is based on a specially developed transfer function that utilizes a modification in a certain frequency range within the acquired pulse signal to derive the aortic pressure wave. The modulus and phase characteristics of the Vasotens transfer function have been published previously [12]. The difference in time between the first wave and the second wave (i.e., the reflected wave) correlates to the distance, according to the manufacturer's instructions, and allows calculation of the pulse wave velocity (PWV). A detailed description of the Vasotens methodology may be found in previous publications [10, 12, 17].

The following indices were derived. The reflected wave transit time (RWTT) represents the transit time of pulse wave along a corresponding artery and is the reciprocal of PWV [10]. In case of a stiff artery, the magnitude of this index is reduced. The method used to estimate this parameter is based on the identification of the reflected wave on the pulse curve in sphygmogram records by original Vasotens algorithm. Since measures of arterial stiffness depend on BP and heart rate (HR) values [1], RWTT is usually normalized to a SBP of 100 mmHg and a HR of 60 bpm by a regression analysis of 24-hour RWTT to 24-hour SBP and 24-hour HR in each individual. PWV indicates the pulse wave speed in the arterial tree: if the artery is stiff, the speed is increased [1]. PWV was also normalized by BP and HR, applying the same methodology used for RWTT. The augmentation index (AI) is defined as the percentage ratio of the pressure increment caused by the reflected wave to the direct wave [1]. Normally, the reflected component in peripheral waves is always smaller than the direct component and AI is negative. In case of high arterial stiffness, the addition of the reflected component caused by different timing may exceed the direct component and the index becomes positive. AI is strongly dependent on HR, so the index is corrected for a HR of 75 bpm as described above. Additionally, AI was calculated from the central waveform reconstructed by transfer function analysis. Finally, we calculated the ambulatory arterial stiffness index (AASI), as one minus the slope of regression of DBP relative to SBP [18].

### 2.4. Statistical Analysis

The analysis of 24-hour BP recordings was preceded by removal of artifacts according to the previously described editing criteria [19]. Recordings were considered valid when at least 70% of expected measurements were available, as recommended by current guidelines [16, 19]. All the BP and arterial stiffness indices estimated in each single BP measurement were averaged for any given subject in order to obtain the 24-hour mean value. Additionally, the daytime and nighttime subperiods were defined according to sleeping times reported in the individual patient's diary cards: average measures for such awake and asleep periods were then computed.

Mean values obtained in each individual subject were averaged for the whole study population, separately for the healthy normotensive and hypertensive group. Differences in hemodynamic indices were assessed by analysis of variance, without adjustment (crude estimate) and after accounting for age, gender, body mass index (BMI), antihypertensive drug treatment, and mean ABP (adjusted estimate). Adjustment for mean ABP was not applied to normalized RWTT and normalized PWV, because these measures were already normalized to a SBP of 100 mmHg and to central BP. Comparison of categorical variables was made by a Chi-square test. To check the relation between the studied parameters, Pearson's correlation coefficient (*r*) was determined. The level of statistical significance was kept at 0.05 throughout the whole study. Data are shown as mean ± SD or as mean and 95% confidence interval for continuous variables and as absolute (*n*) and relative (%) frequencies for discrete variables.

## 3. Results

### 3.1. Demographic and Clinical Characteristics of the Study Population

Overall, 916 people were recruited and performed an ABP recording, of which 182 were healthy volunteers and 734 were hypertensive patients. In the control group, 40 subjects were not considered eligible for inclusion in the analysis because either of ABP recordings did not meet quality criteria (*n* = 10) or office or ABP were elevated. In the hypertension group valid ABP recordings could not be obtained in 73 patients. Thus, in summary, 803 subjects were included in the analysis, of which 142 were controls and 661 were patients with arterial hypertension. The average percentage of valid readings obtained over the 24 hours was 93.9% in the healthy control and 93.1% in the hypertensive group. The average number of valid readings available during the day was 32.9 ± 9.4 in the control group and 26.7 ± 5.6 in the hypertension group, while the corresponding figure for the nighttime period was 7.6 ± 2.6 and 8.2 ± 2.6.

Comparison of the baseline clinical characteristics of the two groups showed that hypertensive patients were older, were more often females, were thinner, had higher office SBP values, and had higher ABP than healthy controls (Table 1). A quarter (24.1%) of the hypertensive patients were regularly taking BP lowering medications.

### 3.2. Ambulatory Central BP and Arterial Stiffness Parameters

Both 24-hour central SBP and DBP were significantly higher in hypertensives than in healthy controls (Table 2). RWTT was significantly lower, whereas aortic PWV and peripheral and aortic AIs were significantly higher, in the hypertension group (Table 2). Also the AASI was significantly higher in hypertensive than in normotensive healthy subjects. After adjustment for confounding factors, a statistically significant between-group difference was still observed for central BP, RWTT, and AI, only.

**Figure 1:**
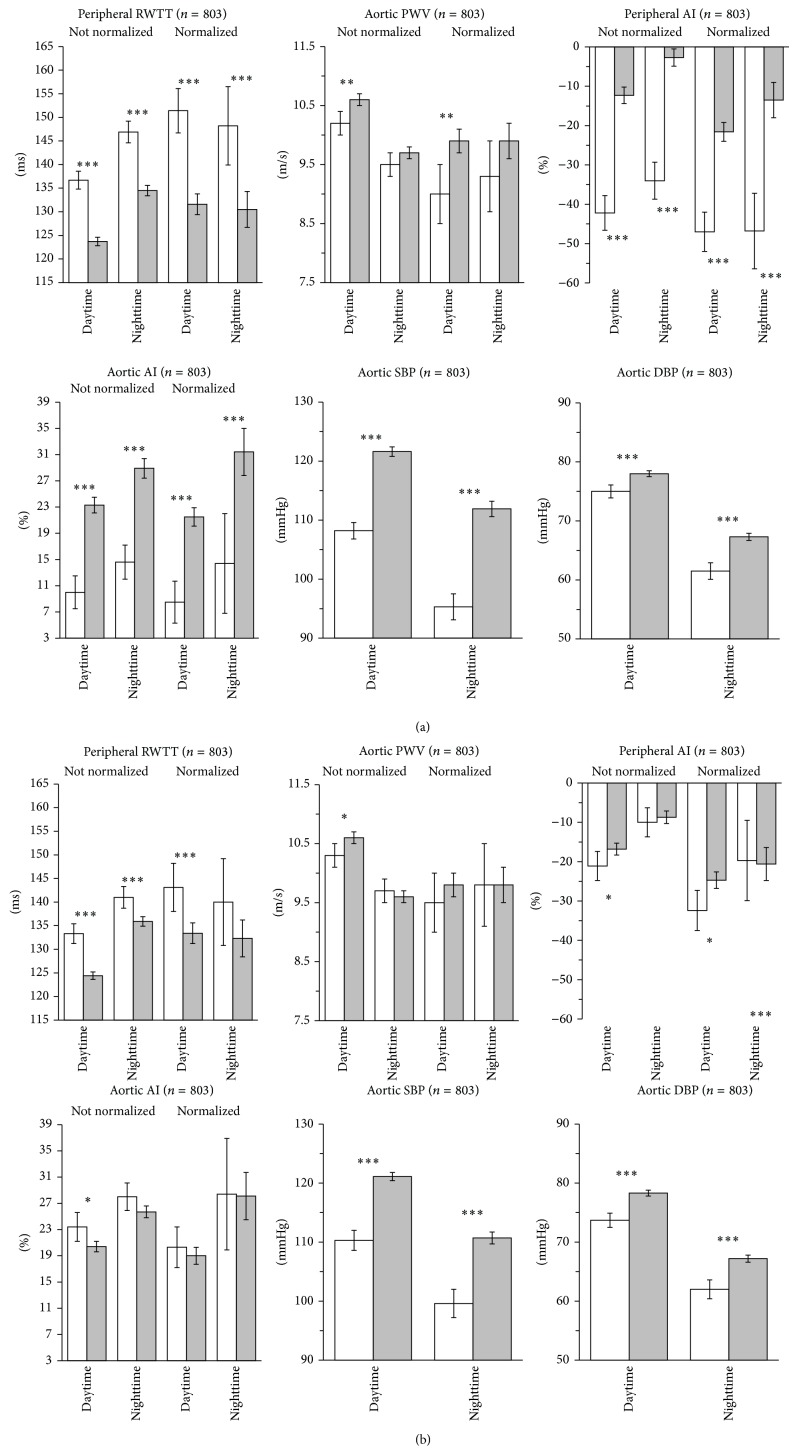
Daytime and nighttime reflected wave transit time (RWTT), aortic pulse wave velocity (PWV), peripheral and aortic augmentation index (AI), and central (aortic) systolic (SBP) and diastolic blood pressure (DBP) values in healthy controls (open bars) and hypertensive patients (gray bars). For RWTT, PWV, and AI not normalized and normalized data (corrected for SBP and/or heart rate) are represented. Data are shown as averages and 95% confidence intervals for crude estimates (a) and for adjusted estimates ((b) data adjusted by age, gender, body mass index, antihypertensive treatment, and 24-hour SBP and DBP). The asterisks indicate the level of the statistical significance of the difference between the two study groups (^***^ *P* < 0.001; ^**^ *P* < 0.01; ^*^ *P* < 0.05).

When indices were assessed separately for the awake and asleep periods, before adjustment, all of them were significantly different between the two groups for both the daytime and nighttime periods, with the exception of nighttime PWV (Figure 1(a)). After adjustment for confounding factors, only RWTT and central BP resulted systematically different between healthy subjects and hypertensive patients for both daytime and nighttime (Figure 1(b)). To note, all estimates of vascular health displayed a typical circadian rhythm: during night sleep RWTT and AI increased, while PWV and central BP decreased. Such a pattern was lost after correcting RWTT and aortic PWV by SBP and HR and peripheral AI by HR (normalized indices).

### 3.3. Correlation between Age, BP, and Arterial Stiffness Parameters

As shown in Table 3, for both healthy subjects and hypertensive patients the relation between age and BP or between age and the different indices of arterial stiffness was statistically significant: the only exception was peripheral SBP and AASI in controls. The magnitude of the correlation coefficient was the highest for AI, with no differences between controls and hypertensives.

As expected, peripheral BP and aortic BP were highly correlated with each other. Conversely, a weak relationship was observed between brachial BP and the different indices of arterial stiffness, though in some cases such a correlation was statistically significant, particularly in the hypertensive group (Table 3).

### 3.4. Correlation between Ambulatory Arterial Stiffness Indices

The different measures of arterial stiffness were variably correlated with each other (Table 4). Regarding central BP, the best correlation was found between peripheral or aortic AI and aortic SBP, with higher values in the hypertensive group. Aortic AI and brachial AI were highly correlated with each other, as was the case with RWTT versus PWV. A poor correlation was observed between PWV and AI and between all arterial stiffness indices and AASI, the only exception being represented by AI in hypertensive patients. In almost all cases correlation coefficients were better in healthy controls.

## 4. Discussion

In this study we report on the absolute levels and circadian pattern of arterial stiffness indices and central hemodynamics evaluated in dynamic conditions over the 24 hours in a large cohort of healthy volunteers and hypertensive subjects. Estimation was based on brachial pulse wave analysis of oscillograms obtained noninvasively by a validated cuff-based BP measuring device. We documented higher peripheral and central BP, higher PWV and AI, and lower RWTT in hypertensive than in normotensive subjects, suggesting that arterial indices derived from oscillometric ambulatory BP measures may help to detect differences in arterial function and to investigate vascular impairment in hypertension. When crude estimates were corrected by confounding factors (age, gender, BMI, and antihypertensive treatment ± 24-hour average BP levels), statistically significant between-group differences were still observed for central BP, RWTT, and AI, only. This suggests that these indices may be unaffected by intrinsic subjects' characteristics and/or BP levels and may thus represent a more sensitive index for evaluating arterial function, at least in ambulatory conditions.

Our paper also provides additional findings which are worth being discussed in detail.

The different indices displayed a typical circadian pattern, regardless of the normotensive or hypertensive status. In particular, central BP followed a diurnal course similar to that of peripheral brachial BP and thus decreased during nocturnal sleep. Conversely, AI increased overnight, likely because this index is inversely related to heart rate, which decreases at night, and it is strongly affected by the body posture, with absolute values increasing during recumbency [20, 21] and decreasing from supine to upright position, irrespective of age, due to a decrease in arterial wave reflection [22, 23].

Standardization of the AI to heart rate removed the diurnal profile, in case of being peripheral but not aortic AI. RWTT and PWV both depend on BP and were, respectively, higher and lower at night than at day. As in the case of peripheral AI, circadian pattern of RWTT and PWV both disappeared after correction for SBP and HR, suggesting that standardized parameters may be more robust as compared to uncorrected ones. All these findings are in line with and confirm those obtained in healthy volunteers or hypertensive patients with other oscillometric devices; although such studies were based on a different technology, they were carried out in smaller groups of subjects and the majority of them evaluated central BP only [11, 24–26]. Our study also adds data to existing evidence collected in normotensive volunteers with the same technology [27]. Interestingly, regardless of the awake or asleep period and of the presence or absence of a circadian rhythm and with the only exception of nighttime PWV, average transit time was lower and PWV, AI, and central BP were higher in hypertensive than normotensive individuals.

We also examined the correlation between the different central and peripheral hemodynamic and arterial stiffness indices. A close relation was found between age and 24-hour arterial stiffness, in both healthy individuals and hypertensive patients, confirming previous evidence collected in resting conditions [28, 29]. ABP was weakly correlated with arterial stiffness indices suggesting that normalized pulse wave analysis may provide an estimate of arterial function involvement independently of BP levels. However, further studies are required in this sense. Though limited in size, the statistically significant correlation between the two main measures of arterial stiffness (PWV and AI) and between peripheral and central AI, found in our study, is consistent with results of published reports [28, 30–37]. However, our study is the first documenting such a relation in ambulatory conditions and in either apparently healthy subjects or hypertensive patients. Finally, PWV was poorly related to AASI, a finding which is in contrast with the results of a recent systematic review and meta-analysis of 51 cross-sectional and longitudinal studies in adults, which reported a good correlation between PWV and AASI [38]. However, unlike our study, in all the studies included in the meta-analysis, PWV was measured in resting and not in ambulatory conditions [38]. Conversely, our results support the evidence of another study which explored the relative importance of the different determinants of the AASI through a previously validated one-dimensional computer model of the arterial circulation applied to 10,000 ABPM simulations [39]. Outcomes of such study suggest that the AASI may not accurately reflect arterial stiffness in ambulatory conditions [39].

## 5. Limitations

The results of the present study must be interpreted also in the context of its limitations. First of all, we assessed arterial parameters noninvasively by applying transfer function analysis to an oscillometric reconstructed waveform rather than via a direct measurement. Indeed, several authors have questioned the goodness of the principle of one-site central BP, PWV, and AI measurements by oscillometry [7, 40, 41]. However, at present, oscillometry is a method that can be easily and conveniently employed for 24-hour monitoring of central hemodynamics, allowing obtaining repeated measurements in daily life conditions with hardly any discomfort to the patient. Additionally, the device used in our study has been properly validated versus alternative algorithms for computing arterial stiffness indexes and central hemodynamics, according to commonly accepted and standardized protocols. All these studies documented a good agreement between the oscillometric cuff-based estimates of central BP, PWV, and AI measured by the BPLab and the established radial tonometry methods [12, 14]. We must acknowledge that all these validation studies were conducted in resting laboratory conditions and not in ambulant subjects. Thus, we cannot exclude that values collected in dynamic conditions might be, at least in part, unrelated to those collected with other devices at rest. We can only rely on studies documenting that the feasibility and reproducibility of noninvasive assessment not only of BP but also of vascular biomarkers derived from the pulse wave analysis of oscillograms by the Vasotens technology are acceptable [10, 14]. Second, absolute data collected in this study may be useful as a reference for indices collected with the same device but may not be used for other ambulatory devices, which are based on different algorithms. Third, our hypertensive subjects were characterized by office DBP values that were on average slightly lower than those observed in healthy individuals, while SBP values were only marginally higher. Thus, our hypertensive population may not be fully representative of the category. Fourth, though based on a large sample of subjects, information provided by our study needs to be corroborated by data collected in future large cohort studies. We need to specifically address arterial stiffness indices and central hemodynamics in extended age ranges, in high-risk hypertensive patients, and in subjects with established target organ damage or comorbidities, such as diabetes. In this regard, a large database of patients evaluated at different centers has been recently established (Vasotens Registry).

## 6. Conclusions

Our results suggest that noninvasive assessment of ambulatory arterial stiffness and central hemodynamics may be feasible and help in assessing the degree of impairment of the arterial tree in hypertensive subjects in daily life dynamic conditions. Such an approach may help unraveling subclinical organ damage or functional changes, for instance, due to an increased sympathetic tone, which are typically associated with hypertension. However, further observations in diverse populations are required before ambulatory assessment of the central hemodynamic variables can make it to the clinical practice. Future studies should validate whether the assessment of noninvasive 24-hour central hemodynamics can provide further information regarding CV risk stratification and target organ damage beyond the 24-hour brachial BP. Additionally, reference values specifically obtained by the BPLab monitor, ideally in prospective studies, are needed. Nevertheless, our data may be used as preliminary diagnostic values of BPLab ABPM additional indices in adult healthy normotensive and hypertensive subjects.

## Figures and Tables

**Table 1:** Demographic and clinical data of healthy subjects and hypertensive patients.

	Healthy subjects (*n* = 142)	Hypertensive patients (*n* = 661)	*P* value
Age (years, means ± SD)	41.8 ± 8.8	58.1 ± 14.9	0.0001
Gender (*n*, %)			
Males	*97* (*68.3*)	*292* (*44.2*)	*0.0001 *
Females	*45* (*31.7*)	*369* (*55.8*)	
Height (cm, means ± SD)	172.6 ± 9.2	170.8 ± 10.2	0.050
Weight (kg, means ± SD)	84.9 ± 16.5	78.6 ± 11.6	0.0001
BMI (kg/m^2^, means ± SD)	28.4 ± 4.6	26.9 ± 2.9	0.0001
Antihypertensive treatment (*n*, %)	0 (0.0)	159 (24.1)	0.0001
Office SBP (mmHg, means ± SD)	122.5 ± 7.5	135.2 ± 11.9	0.0001
Office DBP (mmHg, means ± SD)	78.7 ± 8.0	78.5 ± 8.0	0.807
24-hour SBP (mmHg, means ± SD)	115.3 ± 6.7	129.3 ± 11.6	0.0001
24-hour DBP (mmHg, means ± SD)	71.3 ± 4.9	74.3 ± 6.6	0.0001
Daytime SBP (mmHg, means ± SD)	118.3 ± 6.8	132.1 ± 11.5	0.0001
Daytime DBP (mmHg, means ± SD)	74.0 ± 4.9	76.7 ± 6.8	0.0001
Nighttime SBP (mmHg, means ± SD)	103.1 ± 8.2	120.1 ± 15.7	0.0001
Nighttime DBP (mmHg, means ± SD)	60.9 ± 6.2	66.3 ± 8.6	0.0001

BMI: body mass index; SBP: systolic blood pressure; DBP: diastolic blood pressure.

Data are reported as means ± SD or as absolute (*n*) and relative (%) frequencies. The *P* values refer to the statistical significance of the difference between the two study groups.

**Table 2:** Unadjusted (crude) and adjusted (by age, gender, body mass index, antihypertensive drug treatment, and 24-hour SBP and DBP) estimates for 24-hour BP and arterial stiffness parameters in healthy subjects and hypertensive patients.

	Crude estimate	*P* value	Adjusted estimate	*P* value
	(mean, 95% confidence interval)		(mean, 95% confidence interval)	
Aortic SBP (mmHg)				
Healthy subjects (*n* = 142)	105.6 (103.9, 107.2)	0.0001	108.3 (106.5, 110.0)	0.0001
Hypertensive patients (*n* = 661)	119.3 (118.6, 120.1)		118.7 (118.0, 119.5)	
Aortic DBP (mmHg)				
Healthy subjects (*n* = 142)	72.3 (71.2, 73.4)	0.0001	71.4 (70.2, 72.6)	0.0001
Hypertensive patients (*n* = 661)	75.6 (75.1, 76.1)		75.8 (75.3, 76.3)	
RWTT (ms)				
Healthy subjects (*n* = 142)	139.0 (137.2, 140.8)	0.0001	134.5 (132.5, 136.5)	0.0001
Hypertensive patients (*n* = 661)	126.6 (125.7, 127.4)		127.5 (126.7, 128.3)	
Normalized RWTT (ms)				
Healthy subjects (*n* = 142)	150.7 (146.5, 154.8)	0.0001	142.7 (138.3, 147.2)	0.005
Hypertensive patients (*n* = 661)	133.9 (131.9, 135.8)		135.5 (133.7, 137.4)	
Aortic PWV (m/s)				
Healthy subjects (*n* = 142)	10.0 (9.9, 10.2)	0.007	10.2 (10.0, 10.4)	0.410
Hypertensive patients (*n* = 661)	10.3 (10.2, 10.4)		10.3 (10.2, 10.4)	
Normalized aortic PWV (m/s)				
Healthy subjects (*n* = 142)	9.2 (8.7, 9.6)	0.025	9.5 (9.0, 10.1)	0.617
Hypertensive patients (*n* = 661)	9.8 (9.5, 10.0)		9.7 (9.5, 9.9)	
Peripheral AI (%)				
Healthy subjects (*n* = 142)	−40.7 (−45.1, −36.4)	0.0001	−20.0 (−23.6, −16.3)	0.005
Hypertensive patients (*n* = 661)	−9.7 (−11.7, −7.7)		−14.1 (−15.6, −12.6)	
Normalized peripheral AI (%)				
Healthy subjects (*n* = 142)	−46.8 (−51.8, −41.9)	0.0001	−31.5 (−36.3, −26.6)	0.015
Hypertensive patients (*n* = 661)	−21.3 (−23.6, −19.1)		−24.6 (−26.6, −22.6)	
Aortic AI (%)				
Healthy subjects (*n* = 142)	11.0 (8.5, 13.5)	0.0001	23.4 (21.3, 25.6)	0.252
Hypertensive patients (*n* = 661)	24.7 (23.5, 25.8)		22.0 (21.2, 22.9)	
Normalized aortic AI (%)				
Healthy subjects (*n* = 142)	8.6 (5.5, 11.6)	0.0001	19.7 (16.7, 22.7)	0.742
Hypertensive patients (*n* = 661)	21.5 (20.1, 22.9)		19.1 (17.9, 20.3)	
AASI				
Healthy subjects (*n* = 142)	0.27 (0.24, 0.29)	0.0001	0.38 (0.35, 0.41)	0.172
Hypertensive patients (*n* = 661)	0.42 (0.41, 0.44)		0.40 (0.39, 0.41)	

SBP: systolic blood pressure; DBP: diastolic blood pressure; RWTT: reflected wave transit time; PWV: pulse wave velocity; AI: augmentation index; AASI: ambulatory arterial stiffness index.

Data are shown as averages and 95% confidence intervals. The *P* values indicate the level of the statistical significance of the difference between the two study groups.

**Table 3:** Correlation coefficients of 24-hour BP and arterial stiffness measures with age and peripheral (brachial) SBP and DBP.

	Age (years)		Peripheral SBP (mmHg)		Peripheral DBP (mmHg)	
	Healthy subjects (*n* = 142)	Hypertensive patients (*n* = 661)	Healthy subjects (*n* = 142)	Hypertensive patients (*n* = 661)	Healthy subjects (*n* = 142)	Hypertensive patients (*n* = 661)
Peripheral SBP (mmHg)	0.02	0.22^**^	—	—	0.56^**^	0.21^**^
Peripheral DBP (mmHg)	0.27^**^	−0.13^**^	0.56^**^	0.21^**^	—	—
Aortic SBP (mmHg)	0.28^*^	0.35^**^	0.90^**^	0.96^**^	0.69^**^	0.27^**^
Aortic DBP (mmHg)	0.27^**^	−0.12^**^	0.55^**^	0.22^*^	0.99^**^	0.99^**^
RWTT (ms)	−0.25^**^	−0.35^**^	−0.08	−0.03	−0.11	−0.06
Normalized RWTT (ms)	−0.38^**^	−0.31^**^	0.10	−0.08^*^	0.14	0.03
Aortic PWV (m/s)	0.41^**^	0.26^**^	0.30^**^	0.02	0.31^**^	0.12^**^
Normalized aortic PWV (m/s)	0.44^**^	0.19^**^	0.04	0.08^*^	0.03	0.01
Peripheral AI (%)	0.56^**^	0.62^**^	−0.03	0.28^**^	0.16	−0.11^**^
Normalized peripheral AI (%)	0.39^**^	0.39^**^	−0.08	0.12^**^	0.08	−0.08
Aortic AI (%)	0.57^**^	0.60^**^	0.01	0.33^**^	0.22^**^	−0.11^**^
Normalized aortic AI (%)	0.41^**^	0.41^**^	−0.05	0.18^**^	0.13	−0.08^**^
AASI	−0.05	0.36^**^	0.28^**^	0.53^**^	0.03	−0.19^**^

SBP: systolic blood pressure; DBP: diastolic blood pressure; RWTT: reflected wave transit time; PWV: pulse wave velocity; AI: augmentation index; AASI: ambulatory arterial stiffness index.

Data are separately shown for healthy subjects and hypertensive patients. The asterisks refer to the statistical significance of the correlation coefficient (^**^ *P* < 0.01; ^*^ *P* < 0.05).

**Table 4:** Correlation coefficients between reflected wave transit time (RWTT), pulse wave velocity (PWV), augmentation index (AI), ambulatory arterial stiffness index (AASI), and ambulatory blood pressure (BP) evaluated in the study.

	RWTT (ms)		Aortic PWV (m/s)		Peripheral AI (%)		Aortic AI (%)	
	Healthy subjects (*n* = 142)	Hypertensive patients (*n* = 661)	Healthy subjects (*n* = 142)	Hypertensive patients (*n* = 661)	Healthy subjects (*n* = 142)	Hypertensive patients (*n* = 661)	Healthy subjects (*n* = 142)	Hypertensive patients (*n* = 661)
Aortic SBP (mmHg)	−0.18^*^	−0.06	0.37^**^	0.02	0.34^**^	0.45^**^	0.37^**^	0.50^**^
Aortic DBP (mmHg)	−0.18^*^	−0.11^**^	0.31^**^	0.11^**^	0.20^*^	−0.07	0.26^**^	−0.07
Peripheral RWTT (ms)	—	—	−0.55^**^	−0.63^**^	−0.44^**^	−0.39^**^	−0.51^**^	−0.29^**^
Aortic PWV (ms/s)	−0.55^**^	−0.63^**^	—	—	0.25^**^	0.14^**^	0.29^**^	0.05
Peripheral AI (%)	−0.44^**^	−0.39^**^	0.25^**^	0.14^**^	—	—	0.92^**^	0.89^**^
Aortic AI (%)	−0.51^**^	−0.29^**^	0.29^**^	0.05	0.92^**^	0.89^**^	—	—
AASI	0.08	−0.06	−0.11	0.06	−0.05	0.39^**^	−0.03	0.41^**^

SBP: systolic blood pressure; DBP: diastolic blood pressure.

Data are separately shown for healthy subjects and hypertensive patients. The asterisks refer to the statistical significance of the correlation coefficient (^**^ *P* < 0.01; ^*^ *P* < 0.05).
